# WiseNET: An indoor multi-camera multi-space dataset with contextual information and annotations for people detection and tracking

**DOI:** 10.1016/j.dib.2019.104654

**Published:** 2019-10-16

**Authors:** Roberto Marroquin, Julien Dubois, Christophe Nicolle

**Affiliations:** a Laboratory Image and Artificial Vision (ImViA), University of Bourgogne Franche-Comté, Dijon, France; b Laboratory Connaissance et Intelligence Artificielle Distribuées (CIAD), University of Bourgogne Franche-Comté, Dijon, France

**Keywords:** Indoor multi-camera multi-space dataset, People detection, People tracking, BIM, Contextual information

## Abstract

Nowadays, camera networks are part of our every-day life environments, consequently, they represent a massive source of information for monitoring human activities and to propose new services to the building users. To perform human activity monitoring, people must be detected and the analysis has to be done according to the information relative to the environment and the context. Available multi-camera datasets furnish videos with few (or none) information of the environment where the network was deployed. The proposed dataset provides multi-camera multi-space video sets along with the complete contextual information of the environment. The dataset regroups 11 video sets (composed of 62 single videos) recorded using 6 indoor cameras deployed on multiple spaces. The video sets represent more than 1 h of video footage, include 77 people tracks and captured different human actions such as walking around, standing/sitting, motionless, entering/leaving a space and group merging/splitting. Moreover, each video has been manually and automatically annotated to include people detection and tracking meta-information. The automatic people detection annotations were obtained by using different complexity and robustness detectors, from machine learning to state-of-art deep Convolutional Neural Network (CNN) models. Concerning the contextual information, the Industry Foundation Classes (IFC) file that represents the environment's Building Information Modeling (BIM) data is also provided. The BIM/IFC file describes the complete structure of the environment, it's topology and the elements contained in it. To our knowledge, the WiseNET dataset is the first to provide a set of videos along with the complete information of the environment. The WiseNET dataset is publicly available at https://doi.org/10.4121/uuid:c1fb5962-e939-4c51-bfd5-eac6f2935d44, as well as at the project's website http://wisenet.checksem.fr/#/dataset.

**Table:** Specifications Table

Subject area	*Computer vision*, *BIM*, *IFC*, *deep learning*.
More specific subject area	*Multi-camera multi-space analysis*, *people detection*, *people tracking*.
Type of data	*Videos*, *IFC file of the environment*, *and annotations for people detections and tracking*.
How data was acquired	*The videos were recorded in an indoor multi-space environment*, *using six* Raspberry Pi 3 model B1 with the camera module v2.12.
Data format	*The raw videos are given in a compressed format (*.*avi)*. *The environment information is given in an IFC format (*.*ifc) and the annotations (which result from the video analysis) are given separately in JSON format (*.*json).*
Experimental factors	*The cameras were time synchronized*.
Experimental features	*Not all the videos are scripted*. *The annotations were performed both manually and automatically by using a state-of-the art people detector*.
Data source location	*Institut Marey et Maison de la Métallurgie (I3M) building*, *Dijon*, *France*
Data accessibility	*All the data and scripts are provided in 4TU*.*ResearchData*, https://doi.org/10.4121/uuid:c1fb5962-e939-4c51-bfd5-eac6f2935d44
Related research article	*R*. *Marroquin, J*.*Dubois*, *C*.*Nicolle*, *Ontology for a Panoptes building*: *Exploiting contextual information and a smart camera network*, *Semantic Web 9(6) (2018)*, *803–828* [1]

**Table:** 

**Value of the Data** • The data presented are synchronized video streams which were acquired in a multi-space indoor environment. The proposed data could be used as a benchmark for people detection, as well as for Multi-Target Multi-Camera (MTMC) tracking [[2], [3], [4], [5], [6], [7], [8]], thanks to the given **automatic and manual** annotations as in Ref. [5]. • The WiseNET dataset includes the complete information of the indoor environment, as well as relevant contextual information. This differentiate our dataset to the state-of-the-art ones [[4], [5], [6], [7]]. The environment information is given as an Industry Foundation Classes (IFC) file^1^ that represents the environment's Building Information Modeling (BIM) data. While the contextual information includes a semantic relation between real object (e.g., cameras, spaces) with some enter/exit regions of interest (i.e., doors). • The proposed video sets could be used for human-action recognition such as walking around, standing/sitting, motionless, entering/leaving a space and group merging/splitting. Moreover, they could be also be used for office-objects detections such as tables, monitors, chairs, etc. Furthermore, one camera view only includes shadows of people moving around. • Each frame was timestamped and annotated using a JSON format, making the meta-data easy to read, understand and re-use.

## 1 Data

**Fig. 1:**
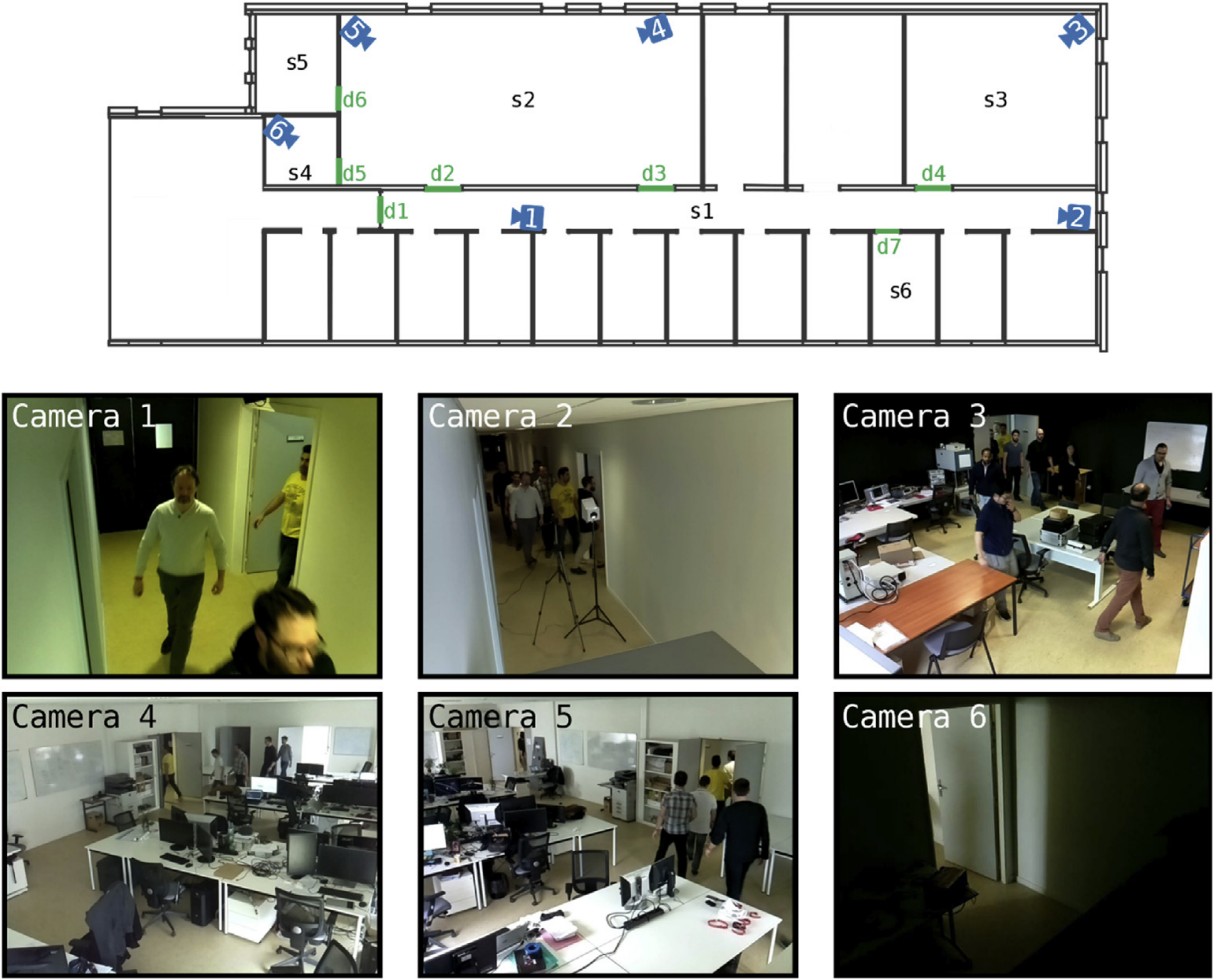
WiseNET network. (Top) Illustration of the position of the camera nodes and the spaces (s1-s6) and doors (d1-d7) of interest. (Bottom) Example images extracted from the video set #5 at different times: at 0′14″ for cameras 1, 4, 5 and 6; and at 0′30″ for cameras 2 and 3.

**Fig. 2:**
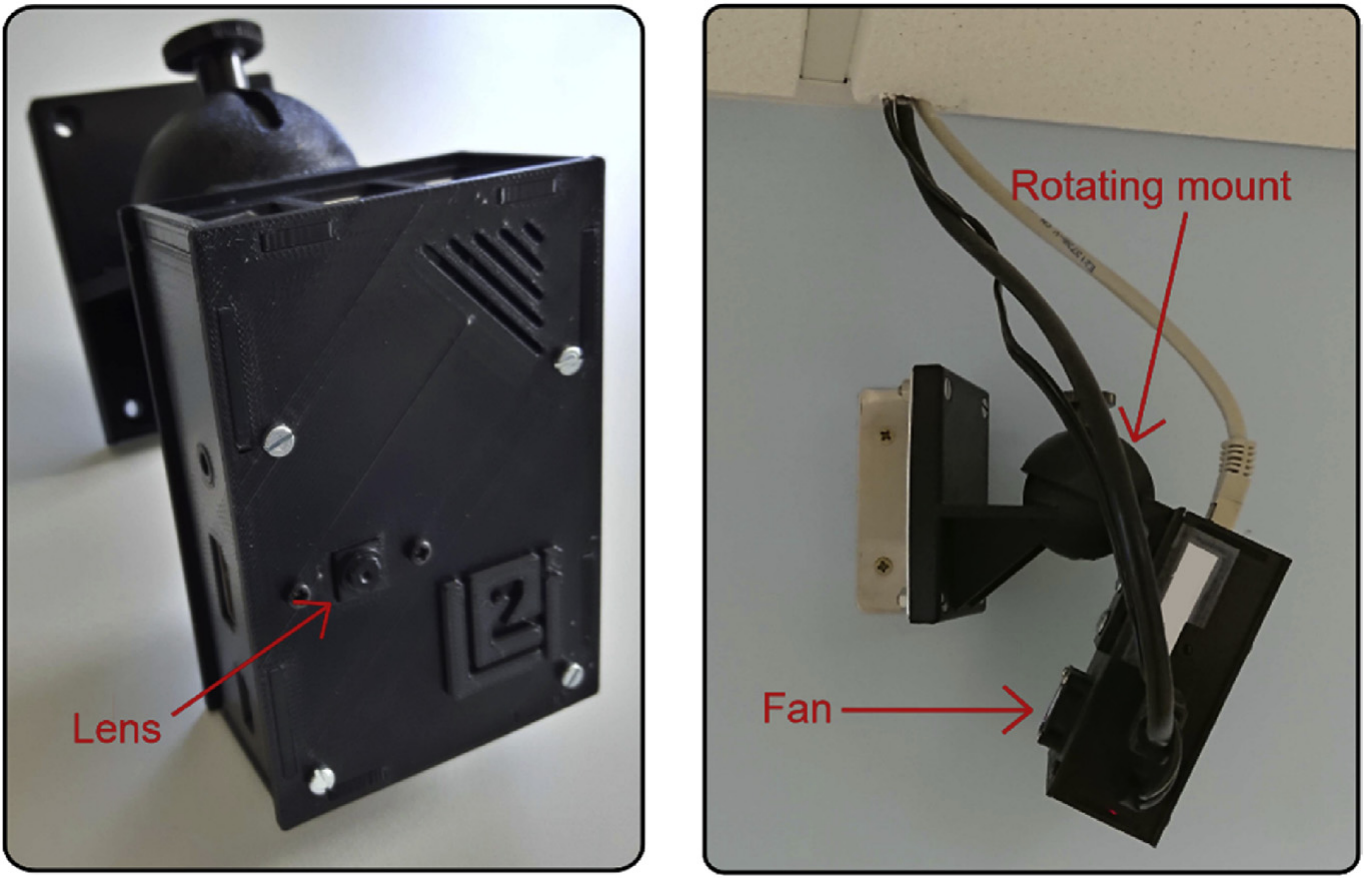
Smart camera based on the Raspberry pi 3 used in the WiseNET network. The camera case was specially designed to include a fan and a rotating mount.

The WiseNET dataset was created using an indoor network composed of 6 smart cameras. The network was deployed on the third floor of the *Institut Marey et Maison de la Métallurgie* (I3M) building located in Dijon, France (see Fig. 1). The smart cameras, presented Fig. 2, have been designed specifically for the experiment to embed the selected processing and enable the synchronization of the different video flows. The dataset consists of three main elements: (1) video sets, (2) information of the environment and context and (3) annotations for people detection and people tracking.

1. The video sets were recorded using from 5 to 6 cameras simultaneously. The features of the 11 sets are described Table 1. The videos captured different human actions such as walking around, standing/sitting, motionless, entering/leaving a space and group merging/splitting. In addition, one view only includes shadows of people moving around.

**Table 1:** Description of video sets. FPS stands for frames per seconds; #Videos indicates the number of cameras used in the set; #Frames (Time) indicates the number of frames and recording time of a complete set; #People indicates the number of people present in the set; #PD-MAN refers to the number of manually annotated people detection bounding boxes.

#Set	Resolution	FPS	#Videos	#Frames (Time)	#People	#PD-MAN
1	1280×720	30	5	87495 (01′00″ × 5)	5	13777
2	1280×720	30	5	17767 (02′00″ × 5)	2	8590
3	1280×720	30	5	17758 (02′00″ × 5)	2	9777
4	1280×720	30	5	35747 (04′00″ × 5)	3	17489
5	640×480	25	6	6000 (00′40″ × 6)	14	11495
6	640×480	25	6	6000 (00′40″ × 6)	6	6545
7	640×480	25	6	6000 (00′40″ × 6)	7	7109
8	640×480	25	6	6000 (00′40″ × 6)	6	4605
9	640×480	25	6	6000 (00′40″ × 6)	8	11520
10	640×480	25	6	6000 (00′40″ × 6)	9	10375
11	640×480	25	6	6000 (00′40″ × 6)	15	10631
			**62**	**122021 (1h13′00″)**	**77**	**111913**

**Fig. 3:**
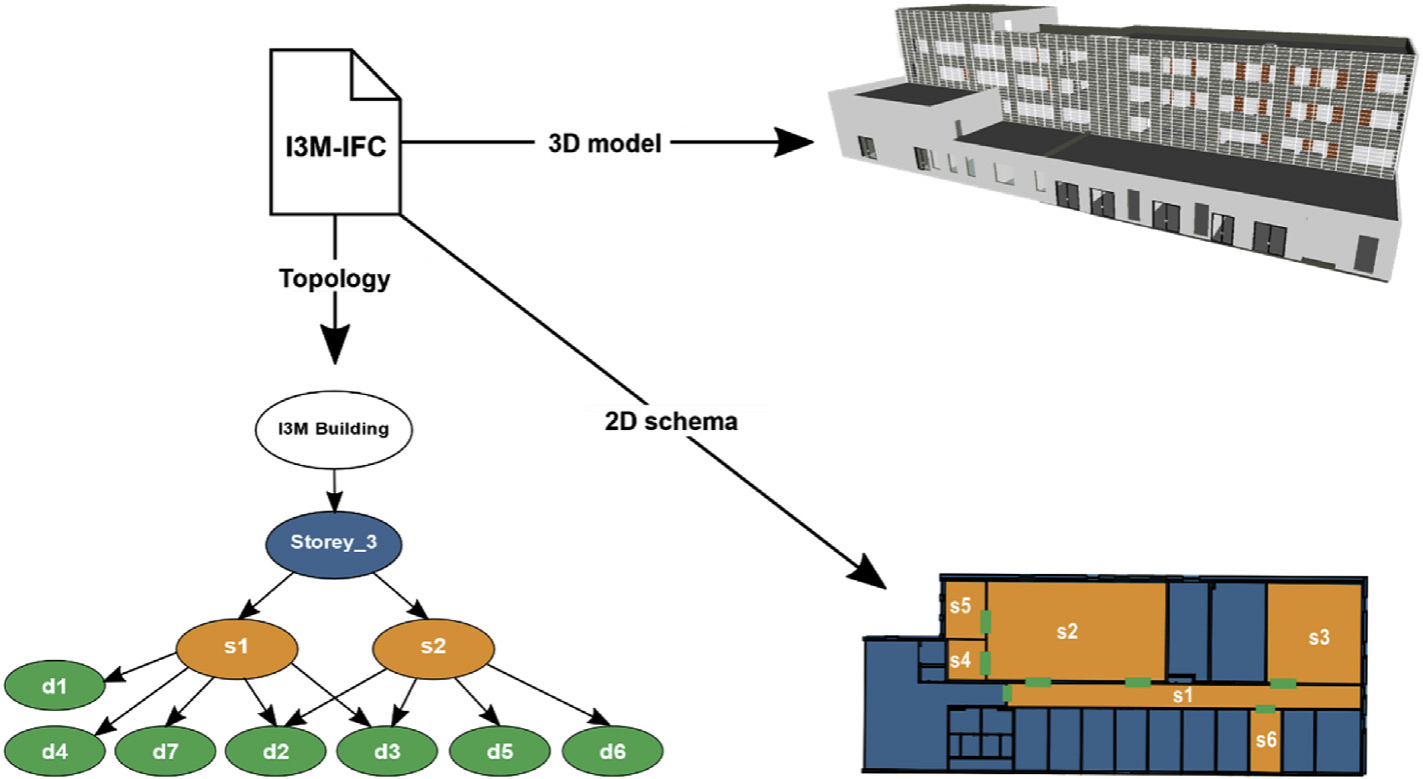
Data generated from the IFC file of the I3M building (I3M-IFC). The 2D schema and the topology graph focus only on the third storey and on some spaces and doors of interest.

2. The dataset includes the IFC file of the I3M building (referred as *I3M-IFC*) and a *camera-calibration* file for each camera node. From the IFC file, different data could be extracted, such as the building's topology and 2D/3D view, as depicted in Fig. 3. Furthermore, the dimensions of the different building elements could also be extracted from an IFC file, as presented in Table 2. Additionally, a camera-calibration file including contextual information is also provided, an example is shown in Listing 1.

**Table 2:** Dimensions of the spaces and doors of interest depicted on Fig. 1. These dimensions were extracted from the IFC file of the I3M building. The elements s1-s6 are spaces and their dimensions are defined as length × width × height. The elements d1-d7 are doors and their dimensions are defined as width × height.

Element	Dimension (m)
s1	27.7×1.747×3.52
s2	14.33×7.04×3.52
s3	7.5×7.04×3.52
s4	2.775×2.91×3.52
s5	3.16×4.14×3.52
s6	2.48×4.47×3.52
d1-d4	1.5×2.075
d5-d7	1×2.075

**Fig. 4:**
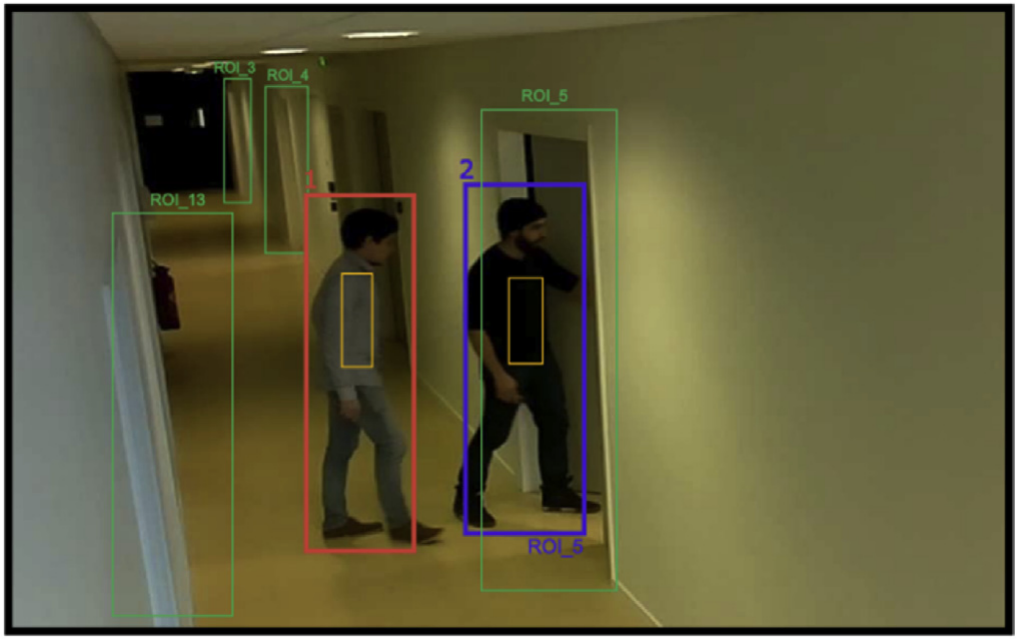
Typical annotated frame of an example of people detection.

**Fig. 5:**
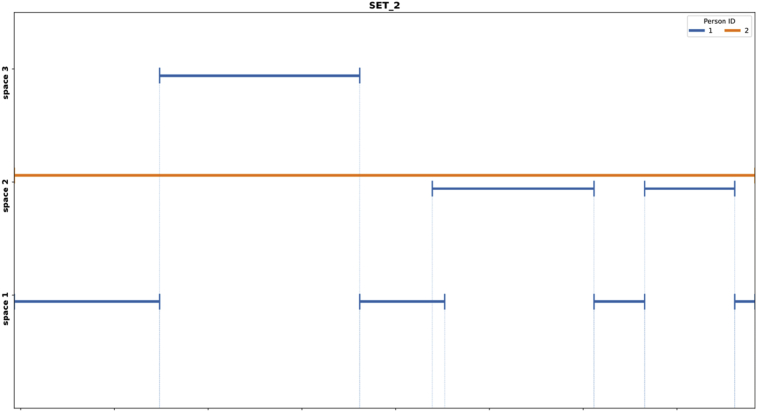
Space-time graph of people tracking ground truth for video set 2.

3. The dataset also includes people detection manual and automatic annotations (PD-MAN and PD-AUT respectively), as well as people tracking manual annotations (PT-MAN). Fig. 4, Fig. 5, present respectively an example of people detection and tracking. The meta-data associated to the detection and tracking, are stored in a JSON structure. Examples of the JSON files are shown in Listings 2 and 3.

**Fig. 6:**
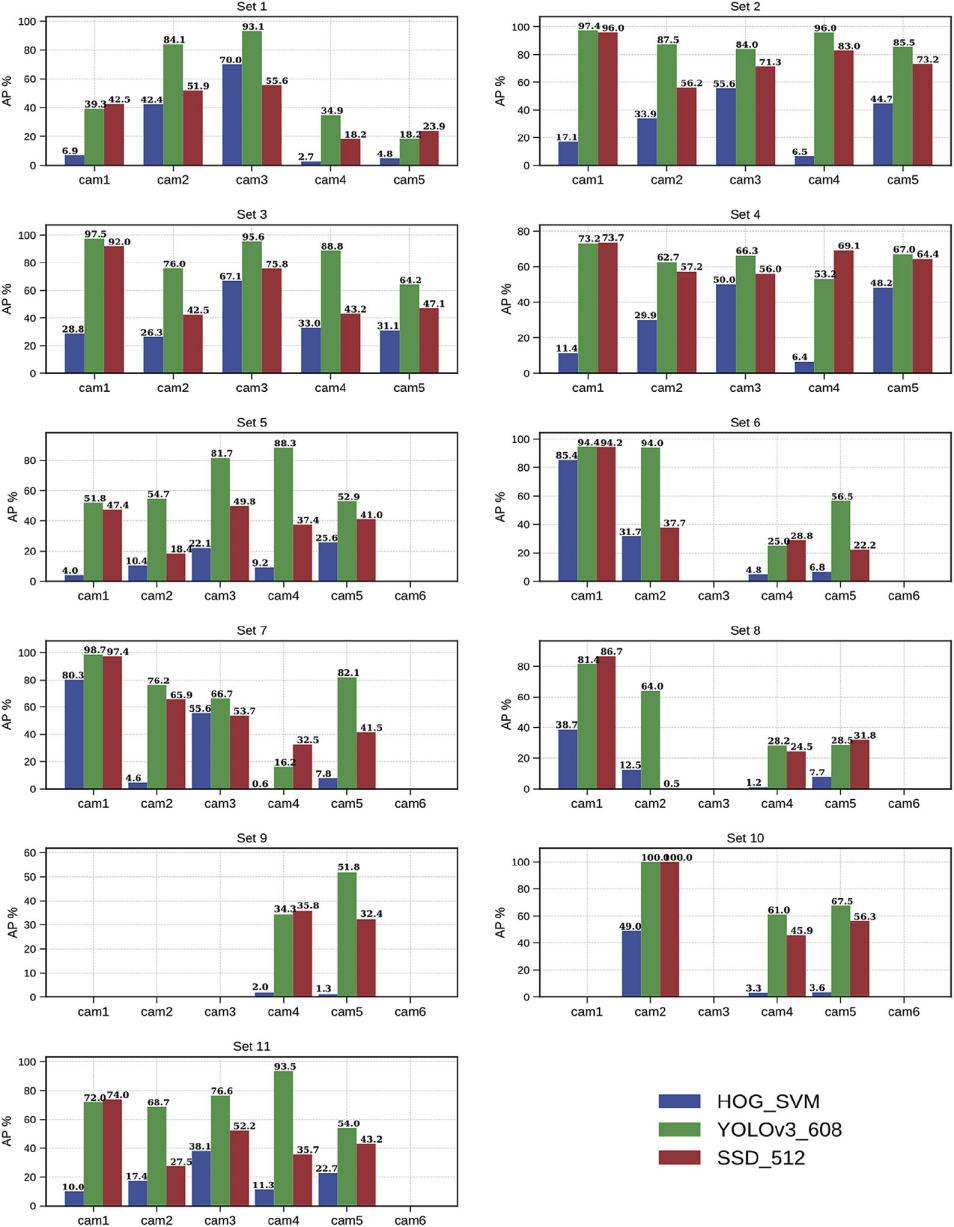
Average Precision (AP) comparison of HOG_SVM [10] (in blue), YOLOv3_608 [12] (in green) and SSD_512 [11] (in red) people detectors, in all the video sets. The videos without detections were ignored.

Furthermore, the use of automatic people annotation aims not only to propose an alternative to the time-consuming manual annotation but also to evaluate the complexity of each video (in terms of difficulty to detect people) using state-of-art people detectors. Therefore, Fig. 6 enables users to select video sets according to their “challenging” level.

## 2 Experimental design, materials, and methods

Fig. 1 presents the distribution of the smart cameras in the environment and some examples of their images. The smart cameras used were the Raspberry Pi 3 model B1 and its camera module v2.12, that contains a Sony IMX219 8-megapixels sensor. A case for the Raspberry Pi was specially designed to include a cooling system and a rotating mount that facilitates its installation, as shown in Fig. 2. The cooling system is important to enable the Raspberry Pi to record for long periods of time without overheating. All the videos were timestamped, and the network was synchronized by implementing a network time protocol (NTP) server [9].

### 2.1 Video sets

Eleven video sets were recorded at different times. A description of each video set is presented on Table 1. In summary, there are 11 video sets, composed of 62 videos that cover more than 1 hour of video footage, 122K frames, 77 people tracks,^2^ around 112000 PD-MAN annotations (details about the annotation procedure are presented in Section 2.3). The video sets were captured at two resolutions—HD 720 (1280×720) or VGA (640×480)—different frames per second (FPS)—30 or 25—various recording time—40 seconds or 1, 2 and 4 minutes—and using two video codecs—MPEG-4 and Planar 4:2:0 YUV. The different recording characteristics lead to a richer and more diversified dataset.

### 2.2 Contextual information

The contextual information of the WiseNET network is composed of two parts, the information of the environment where the network was deployed and the information concerning the camera nodes.

The information about the environment is contained in the I3M-IFC file. IFC is a data representation standard, developed by the buildingSMART,^3^ used to define architectural and construction-related data and to facilitate interoperability between the different agents involved in a building construction. The I3M-IFC contains large amount of information concerning the I3M building, e.g., information about all the elements composing the building, their geometrical information, their position and their relation to other elements. Fig. 3 shows some examples of data that can be generated from the I3M-IFC file. The environment's topology refers to the following tree structure: a building has a set of storeys, the storeys have a set of spaces and the spaces have a set of elements (e.g., doors, windows and sensors). Another example of data that can be obtained from an IFC file are, the dimensions of the spaces where the cameras were installed and the dimensions of the doors they observe, as presented in Table 2. However, to extract information from an IFC file is not an easy task. A way to easily handle the IFC information is by converting the IFC file into an ontology, as presented in Ref. [1] in order to obtain the building's topology.

The I3M-IFC file was obtained from the company in charge of the construction of the I3M.

**Remark** (ID of elements of interest). The dataset includes a file containing the ID of each element of interest, used for extracting (from the I3M-IFC file) the data presented in Table 2.

The *camera-calibration* files contain the position of the camera nodes in the environment and the information about the objects of interest observed by them.

These files are structured in a JSON format^4^ where each field corresponds to:

• *devide ID*: identification of the camera node.
• *is Hosted By*: space where the camera node is located.
• *resolution*: camera's resolution.
  ○ *width*: camera's field of view (FOV) width.
  ○ *height*: camera's FOV height.
• *regions Of Interest*: information about the regions of interest (ROIs) observed by the camera.
  ○ *region Of Interest* (in singular): identification of the ROI
  ○ *xywh*: position of the ROI in the camera's FOV, where (*x*,*y*) are the coordinates of the ROI's top-left point, and (*w*,*h*) are the le width and height respectively.
  ○ *represents*: real object represented by the ROI.

An example of a camera-calibration file is presented in Listing 1. The information contained in the calibration-file can be summarize as: The *Smart Camera 4* is located at the space *s2*, it has a resolution of 1280×720, and it observes three ROIs which represent the doors *d2*, *d5* and *d6*.

Moreover, the selection of ROIs in the camera image is known as *semantic-labelling* and its goal is to relate real objects or important space-regions, with their projections in the camera view. In the WiseNET dataset only doors were considered as ROIs due to their importance in a building environment, e.g., they connect two spaces and people have to pass through them to enter/exit a space.

Listing 1 Camera-calibration file for the Smart Camera 4.

**Figure:**
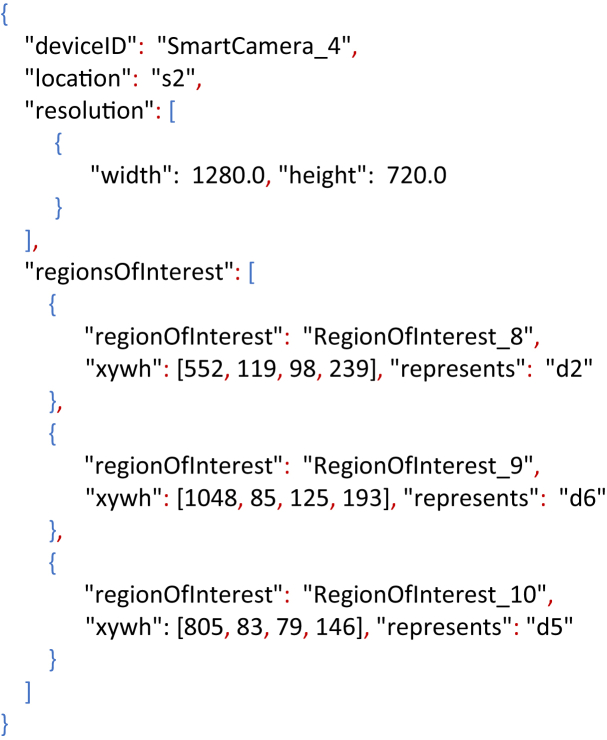

### 2.3 People detection annotations

The people detection manual annotations (PD-MAN) were obtained by manually enclosing a bounding box (Bbox) around each person that appears in a video frame, assigning them a unique identifier (ID) and stating if they are around a ROI. The process was performed by using a software developed in Python^5^ using OpenCV library^6^—the code is provided but not supported. The PD-MAN annotation rules were as follows:

1. On each video set, a unique ID should be associated to each person. For example, the person “Mario” should have the same ID in the five videos composing the set 1. Moreover, if “Mario” appears in another set he might be assigned a different ID.
2. The Bbox should be created by selecting its top-left and bottom-right corners.
3. If only a person's limb is visible, then no Bbox should be drawn.
4. If a person is partially occluded, then the Bbox should enclose only the visible parts.
5. If a person torso is no visible, e.g., only its head is visible, then no Bbox should be drawn.
6. If a person is not visible for the human eye—because is totally occluded by an object, is outside the cameras FoV or the scene is too dark—then no Bbox should be drawn. Even if the person's position could be deduced from previous frames.
7. A person is considered around a ROI if: (1) the center of its Bbox is inside the ROI and (2) if the Bbox is at the same level than the ROI, i.e., if the lowest point of the Bbox and the ROI are around the same height.

**Remark** (Person around ROI). This information not only relates a person with an element of the environment, but also can be used to determine if a person is entering/leaving a space or to help people re-identification between multiple cameras, as done in Ref. [1].

Manual annotation is a time-consuming task; therefore, it was only performed on every fifth frame starting from frame 0. However, the information was propagated to the missing frames, e.g., the annotation in frame 0 was propagated into frames 1, 2, 3 and 4, the same for the annotation in frame 5 and so on.

The PD-MAN annotation can be used to evaluate people detection algorithms, as well as people re-identification—by considering the unique ID.

The use of automatic people annotation aims not only to propose an alternative to the time-consuming manual annotation but also to evaluate the complexity of each video (in terms of difficulty to detect people) using state-of-art people detectors. The people detection automatic annotations (PD-AUT) were obtained by passing each video frame through a set of pre-trained people detector models. We used the well-known people detector Histogram of Oriented Gradients (referred as HOG_SVM), as well as two state-of-the-art CNN-based object detector models: Single Shot Detector (referred as SSD_512) and the You-Only-Look-Once version 3 (referred as YOLOv3_608).

• The HOG_SVM detector is based on HOG feature descriptors and Support Vector Machine (SVM) in order to detect people [10]. We used the implementation provided by the OpenCV library. We chose this detector due to its low complexity which results in a very low processing time.
• SSD_512 is a one-stage detector that extracts the feature map of the complete image, then applies a sequence of multi-scale convolutional layers and anchor boxes in order to classify the different regions of the feature map [11]. We used the pre-trained model—configuration and weights—provided by the authors.^7^ Specifically, we used the model with input image size of 512×512 which was first trained on the COCO dataset (Common Objects in Context)^8^ and then fine tune on the union of PASCAL VOC2007 and VOC2012 dataset.^9^ We chose this detector due to its high precision [11].
• YOLOv3_608 uses a single neural network that predicts bounding boxes and class probabilities directly from full images. We used the pre-trained model—configuration and weights—provided by the authors.^10^ Specifically, we used the model with size 608×608 which was trained on the COCO dataset. We chose this detector due its high precision and low inference time [12], which are two major factors for a real-time surveillance system.

For the object detectors—SSD and YOLO—we only focus on the person class, i.e., the rest of objects were simply ignored. The process was performed by using a software developed in Python using OpenCV library—the code is provided but not supported. The choice of detectors differs in complexity and robustness, which we consider an interesting factor for evaluating the limitations of systems. Moreover, due to the automatic nature of the annotations, the rules presented for the PD-MAN cannot be considered, only the rule stating if a detection is around a ROI is considered (rule 7).

The PD-AUT where obtained for all the frames.

The resulting meta-data from the PD-MAN and PD-AUT annotations were stored using a JSON structure based on the logic that a video has a set of frames and some of those frames present a set of Bboxes (detections). A typical annotated frame is shown on Fig. 4 and the resulting meta-data is the Listing 2. The JSON fields correspond to:

• *video*: name of the video file from which the meta-data was obtained.
• *resol*u*tion*: video resolution.
• *frames*: set of frames with BBoxes. The frames with no BBoxes are not considered.
  ○ *frameNumber*: frame number.
  ○ *deviceID*: ID of the smart camera observing the scene.
  ○ *inXSDDateTime*: time stamp obtained from the smart camera.
  ○ *detections*: set of BBoxes (detections) made in the same frame.
    ⁃ *class*: detection's class name. In our case “person”.
    ⁃ *imageAlgorithm*: Algorithm used for detecting. For example, “YOLO” or “SSD”. If it is a manual annotation, then the value is “groundtruth”.
    ⁃ *xywh*: detection's top-left point (x; y), width (w) and height (h).
    ⁃ *id*: person's ID.
    ⁃ *regionOfInterest*: ROI's ID. If the detection is not around a ROI then the value is “null”.
    ⁃ *visualDescriptors*: array of features describing the detection.

Notice that *any type* of visual features can be used to describe visually the detection. For all the PD annotations in the dataset we decided to use *a localize 2D Hue-Saturation (HS) histogram* as visual descriptor $Vd_{m}$, where m is the size of the array and is defined by the number of bins in each channel as $m=H_{n}\times S_{n}$ . We used 9 bins per channel ($H_{n}=S_{n}=9$) for PD-MAN which gives a visual descriptor of 81 features; and 8 bins per channel for PD-AUT which gives a descriptor of 64 features. In the PD-AUT we decided to use 8 bins instead of 9 because in future works, we plan to combine them with other types of visual features and to have 64 features allow us to easily give equal weight to all the types. Finally, the visual descriptor Vd was normalized using the $\ell^{2}-norm$ (see Eq. (1)) in order to keep the relative contribution of the histogram bins regardless of their absolute contribution.

(1) $$\begin{array}{c} \hat{Vd}=\frac{Vd}{\sqrt{\sum_{k=1}^{m}{\left|Vd_{k}\right|}^{2}}} \end{array}$$

Moreover, to avoid the inclusion of the background (i.e., non-informative content) in the visual descriptor, the histogram was computed only from a t-shirt region *not* from the complete detection region, i.e., the *localization* of the histogram plays the role of background subtractor. The t-shirt region was defined by the following equations: $T_{x}=x+T_{w}$ , $T_{y}=y+\left(h\times \alpha \right)$ , $T_{w}=w/3$ , and $T_{h}=h/4$, where $\left(T_{x},\;\;T_{y}\right),\;\;T_{w}$, and $T_{h}$ are the t-shirt's top-left corner coordinates, width and height respectively; (x,y),w, and h are the detection's top-left corner coordinates, with and height respectively; and α is factor that determines the height of the t-shirt region according to the visible body parts (i.e., full body or only torso) and on the position of the body (i.e., profile or front). The factor α is defined as:

(2) $$\begin{array}{c} \alpha =\{\begin{array}{cc} 0.58\;if\;\frac{w}{h}>0.55\; & \left(torso-profile\right)\\ 0.33\;if0.4\leq \frac{w}{h}<0.55\; & \left(torso-front\right)\\ 0.25\;if\;\;0.2\leq \frac{w}{h}<0.4 & \left(full\;body-profile\;\right)\\ 0.2\;if\;\frac{w}{h}<0.2 & \left(full\;body-front\right) \end{array} \end{array}$$

**Remark** (Change of visual descriptor). The manual and automatic annotations **are not** dependent of our choice of visual descriptor and background subtraction. We are providing the video frames and the detections bounding boxes; therefore, the user can feel free to only use the provided bounding boxes and extract any desired visual features.

Listing 2 Extract of a meta-data stored in a JSON structure associated to the typical annotated frame displayed in Fig. 5.

**Figure:**
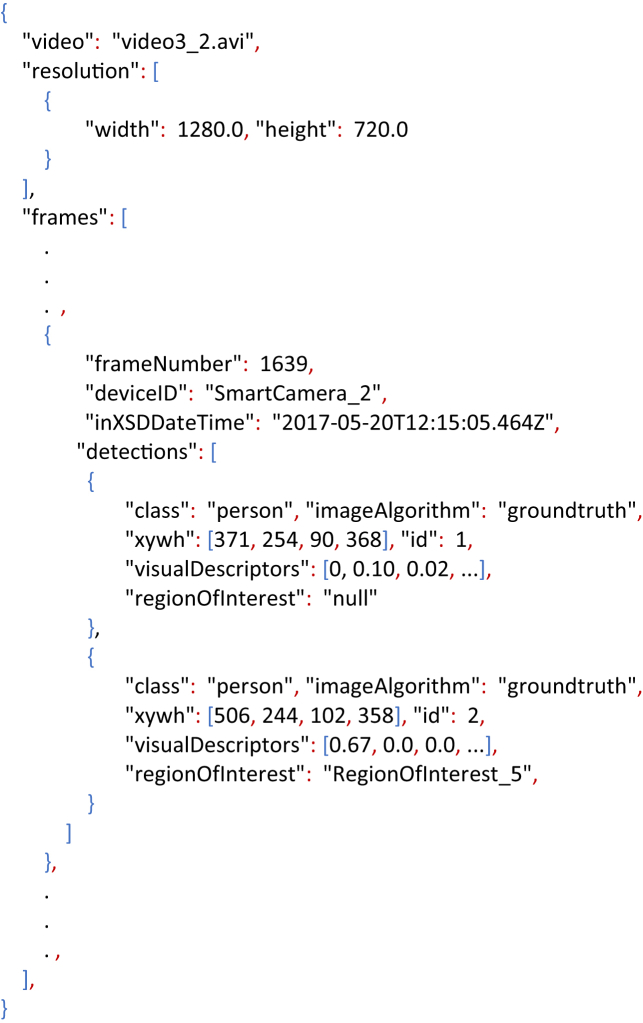

### 2.4 People tracking annotations

People tracking manual annotations (PT-MAN) consisted in manually stating the space location of each person at all times, during a complete video set. This was done by considering the people's ID and the time they enter and leave each space. For each video sequence, the tracking information is given in the form of a space-time graph along with its meta-data stored in a JSON file. The space-time graph is an intuitive way of presenting the location and the changes of spaces of all people during a period of time. The tracking meta-data is stored in a JSON file, where the fields corresponds to:

• *set*: video set number from which the PT-MAN was obtained.
• *tracks*: set of people tracks. A track relates a person with a set of spaces at some periods of time. A track is divided into a set of tracklets.
  ○ *id*: person's ID.
  ○ *tracklets*: set of tracking segments of a person.
    ⁃ *location*: tracklet's space location.
    ⁃ *start*: tracklet's starting time.
    ⁃ *end*: tracklet's end time.

Fig. 5 shows an example of the space-time graph for the video set 2. Its meta-data stored in a JSON structure is presented in Listing 3. From the space-time graph it can be observed that there were 2 people present during the recording; and that *person 1* moved between spaces 1, 2 and 3, while *person 2* stayed at space 2 during the whole recording time. The meta-data file can be used for evaluating the tracking algorithms, for example by using the multi-target multi-camera metrics proposed by Ref. [8].

Listing 3 Extract of the tracking meta-data stored in a JSON structure related to the space-time graph depicted in Fig. 5.

**Figure:**
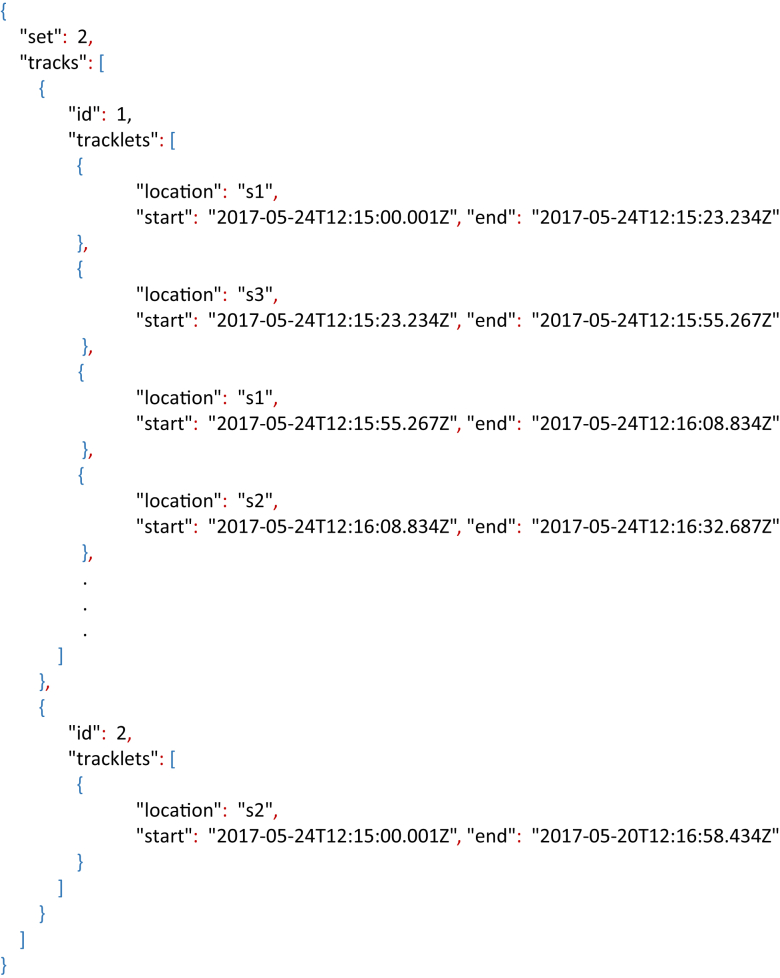

### 2.5 Experimental validation

The experimental evaluation is proposed to validate the usability and quality of the video sets. For this, we used three automatic people detectors—HOG_SVM, SSD_512 and YOLOv3_608— and we evaluate their performance with respect to the PD-MAN annotations, which were consider as ground truth. The following results and the associated analysis aim to demonstrate that each video represent a challenge for people detection and therefore can be used to benchmark the multi-view people tracking.

The metrics used for the evaluation were the *Precision × Recall curves* (PR-Curves) from which the *Average Precision* (AP) can be obtained by computing the area under the curve. These metrics were proposed by the Pascal VOC challenge [13]. We use the Python implementation proposed by R. Padilla with an Intersection Over Union (IOU) threshold of 50%.^11^ Moreover, to use this implementation a script that extracts each detection in the JSON file and convert it to a text file was developed and is also provided.

AP is a numerical metric, which simplifies the comparison of different detectors. Fig. 6 presents a comparison of the resulting APs for all videos in the dataset. The dataset provides all the PR-Curves from which the AP were computed. Notice that the videos without detection (i.e., nobody appeared in the camera's view) were ignored during the evaluation (e.g., the videos from camera 6 in sets 5–11). From the AP, is possible to evaluate the difficulty/challenge degree of each video in the dataset.

It is important to notice that the results presented depend on the quality of the ground truth, which was done by multiple humans, thus is prompt to subjectivity and errors. Moreover, there is some discrepancy between PD-MAN rules and the automatic annotations especially when the person torso is not visible (rule 5), which occurs when the person is much closed to the camera. Furthermore, to obtain the PD-MAN annotations is a time-consuming task. Therefore, for all those reasons we recommend the users of the database to use (if possible) the automatic detections instead of the manual, especially the YOLOv3_608 detections.

**Remark** (Camera 6). Even though camera 6 did not record any person in the sets, it recorded shadows of people walking around space *s2* (see Fig. 1). Thus, we decided to include the videos.

## Footnotes

2 Each people that appear in the video sets signed informed written consent before participating.

3 http://www.buildingsmart-tech.org/specifications/ifc-overview.

4 JSON (JavaScript Object Notation) is a lightweight format to serialize structured data https://www.w3schools.com/js/js_json_syntax.asp.

5 https://www.python.org/.

6 https://opencv.org/.

11 https://github.com/rafaelpadilla/Object-Detection-Metrics.

## Footnotes

1 https://www.iso.org/standard/70303.html.

7 https://github.com/chuanqi305/ssd.

8 http://cocodataset.org/.

9 http://host.robots.ox.ac.uk/pascal/VOC/.

10 https://github.com/pjreddie/darknet/blob/master/cfg/yolov3.cfg.
